# Generating testable hypotheses for schizophrenia and rheumatoid arthritis pathogenesis by integrating epidemiological, genomic, and protein interaction data

**DOI:** 10.1038/s41537-017-0010-z

**Published:** 2017-02-24

**Authors:** Tulsi A. Malavia, Srilakshmi Chaparala, Joel Wood, Kodavali Chowdari, Konasale M. Prasad, Lora McClain, Anil G. Jegga, Madhavi K. Ganapathiraju, Vishwajit L. Nimgaonkar

**Affiliations:** 1 Department of Psychiatry, University of Pittsburgh, Pittsburgh, USA; 2 Department of Biomedical Informatics, School of Medicine, University of Pittsburgh, Pittsburgh, PA USA; 3 Division of Biomedical Informatics, Cincinnati Children’s Hospital Medical Center, Cincinnati, OH USA; 4 Department of Human Genetics, Graduate School of Public Health, University of Pittsburgh, Pittsburgh, USA

## Abstract

Patients with schizophrenia and their relatives have reduced prevalence of rheumatoid arthritis. Schizophrenia and rheumatoid arthritis genome-wide association studies also indicate negative genetic correlations, suggesting that there may be shared pathogenesis at the DNA level or downstream. A portion of the inverse prevalence could be attributed to pleiotropy, i.e., variants of a single nucleotide polymorphism that could confer differential risk for these disorders. To study the basis for such an interrelationship, we initially compared lists of single nucleotide polymorphisms with significant genetic associations (*p* < 1^e-8^) for schizophrenia or rheumatoid arthritis, evaluating patterns of linkage disequilibrium and apparent pleiotropic risk profiles. Single nucleotide polymorphisms that conferred risk for both schizophrenia and rheumatoid arthritis were localized solely to the extended HLA region. Among single nucleotide polymorphisms that conferred differential risk for schizophrenia and rheumatoid arthritis, the majority were localized to *HLA-B*, *TNXB*, *NOTCH4*, *HLA-C*, *HCP5*, *MICB*, *PSORS1C1*, and *C6orf10*; published functional data indicate that HLA-B and HLA-C have the most plausible pathogenic roles in both disorders. Interactomes of these eight genes were constructed from protein–protein interaction information using publicly available databases and novel computational predictions. The genes harboring apparently pleiotropic single nucleotide polymorphisms are closely connected to rheumatoid arthritis and schizophrenia associated genes through common interacting partners. A separate and independent analysis of the interactomes of rheumatoid arthritis and schizophrenia genes showed a significant overlap between the two interactomes and that they share several common pathways, motivating functional studies suggesting a relationship in the pathogenesis of schizophrenia/rheumatoid arthritis.

## Schizophrenia and rheumatoid arthritis: Exploring the link

Researchers in the USA identify variations in eight genes that potentially confer differential risk for schizophrenia and rheumatoid arthritis. Previous studies have shown that patients with schizophrenia (and their relatives) have a reduced risk of developing rheumatoid arthritis and vice versa. Vishwajit Nimgaonkar, Madhavi Ganapathiraju and colleagues at the University of Pittsburgh compared single nucleotide variations in the genetic code that are significantly associated with these diseases. They found that the inverse risk between these disorders could be due to differences harbored in eight genes. Computational analyses of the protein interactomes of genes associated exclusively with rheumatoid arthritis and those with schizophrenia revealed common interacting partners that are known to be involved in immune and inflammatory responses, and shared disease pathways. Additional investigation of these proteins could shed further light on shared disease mechanisms.

## Introduction

Schizophrenia (SZ) is a severe psychiatric disorder of unknown etiology with a lifetime risk of approximately 1%. The heritability of SZ, estimated at ~70%, is best explained by a multi-factorial polygenic threshold model (MFPT) that invokes multiple genetic risk factors modified by the environment.^1^ Ongoing genome-wide association studies (GWAS) of SZ indicate over 100 SNPs and relatively rare mutations of variable effect sizes, but for majority of the loci the primary risk variants and the functions are uncertain.^2^ Further clarity may be gained by an integrative analyses of genomic and proteomic data.^3^

Rheumatoid arthritis (RA) is an autoimmune disease that causes inflammation of the small joints in the hand and feet with a prevalence of approximately 1%. Like SZ, the etiology of RA also is best explained by an MFPT model.^4^ Several studies over the past five decades indicate an inverse prevalence of RA and SZ, i.e., individuals with SZ are less likely to be diagnosed with RA, and vice versa*.* ^5–7^ Children and siblings of individuals with SZ show reduced risk of seronegative RA,^8^ though some reports differ.^5^ The inverse prevalence of RA in patients with SZ and the reduced familial risk of RA raise the possibility that there are shared pathogenic processes for these disorders. A portion of the genetic risk factors could even have pleiotropic effects, i.e., one allele confers risk for SZ, while another variant of the same polymorphism elevates risk for RA. The latter possibility was initially tested in a relatively small under-powered candidate gene studies and GWAS analyses that did not provide any supportive evidence.^9^ Recently, an analysis of larger datasets indicated a small but statistically significant negative correlation between SZ and RA, with stronger effect for SNPs localized to coding and regulatory regions (correlation of −0.046 and −0.174, respectively); these analyses are supported by genetic correlations of a similar magnitude even with GWAS score statistics in lieu of individual-level genotype data.^10^ Thus, it may be worthwhile to search for individual genetic risk factors with pleiotropic effects on SZ/RA.

In a broader framework, the epidemiologic data also motivate a search for shared pathogenic processes. Interactome analysis is a useful approach for discovering novel functional associations of genes. In the present study, we sought individual SNPs with plausible pleiotropic risks and explored their functions through in silico analyses, including interactome analyses, to search for links to shared pathology. We identified several leads that could motivate further hypothesis-driven functional studies.

## Results

To study the molecular and genetic interrelation between rheumatoid arthritis and schizophrenia, we carried out two parallel and independent analyses. The first one was to identify the SNPs that potentially have pleiotropic effects, and the second one was to study the protein interactomes of the genes associated with the two diseases. For the parallel (independent) interactome analyses, we computed novel protein–protein interactions (PPIs) using high-confidence protein–protein interaction prediction (HiPPIP) method that we previously developed.^11^ Additionally, to accelerate future studies of functional mechanisms of genes containing pleotropic SNPs, we also computed their novel PPIs.

### Identification of risk SNPs with apparent pleiotropic effects

The following cascaded analysis of SNPs associated with RA and SZ was carried out to identify pairs of SNPs with strong linkage disequilibrium (LD), such that they conferred risk in opposite directions for the two disorders. A search for pairs of SNPs (*r, z*) where *r* is significantly associated with RA and *z* with SZ, and r and z are in strong LD, yielded 1376 pairs of SNPs; *all* of which were located in the extended HLA region in chromosome 6p. Of these, 46 pairs were identified as having alleles that conferred risk in opposite directions for RA and SZ (Supplementary File 1). Of these, 29 pairs consisted of SNPs with putative *pleiotropic* effects (i.e, *r* and *z* were found to refer to the *same* SNP but with opposing odds ratios in RA versus SZ GWAS), 18 of which were located within gene regions including exonic, intronic and flanking regions, and 11 were not within gene regions using these criteria (see Methods). These 18 SNPs were located to the following 8 genes: *TNXB, HLA-B, HLA-C, NOTCH4, HCP5, MICB*, *PSORS1C1* and *C6orf10* (Table 1)*.* We refer to these 8 as Genes Associated with Putative Pleiotropic SNPs (GAPPS). Four of these genes contain SNPS with pleiotropic effects in their exonic regions: rs915894 in *NOTCH4*, rs2073045 in *C6orf10*, rs1050420 in *HLA-C* and rs709055, rs12721827, and rs1131163 in *HLA-B*.

**Table 1:** 18 putative pleiotropic SNPs

SNP name	SNP Alias	Chr	Base Pair Position (hg19)	Gene	Exon/intron/ flanking region	Allele	RA OR	RA *p*-value	SZ OR	SZ *p*-value
rs3130564	rs3130564	6	31101674	PSORS1C1	intron	T/C	1.23	4.60E-29	0.92969	1.01E-07
rs1050420	rs116597504	6	31239518	HLA-C	exon	T/C	0.9	1.00E-13	1.0709	3.87E-08
rs2524084	rs114208039	6	31241639	HLA-B	intron	A/G	0.83	1.40E-43	1.06855	3.94E-07
rs1634791	rs114802770	6	31276777	HLA-B	intron	A/G	1.15	8.60E-19	0.9366	1.70E-07
rs9265341	rs142174523	6	31294290	HLA-B	intron	A/G	1.32	3.50E-36	0.92663	3.70E-07
rs9265451	rs116690305	6	31296529	HLA-B	intron	A/G	1.3	1.90E-09	0.9209	5.46E-08
rs6902116	rs114876567	6	31300286	HLA-B	intron	A/G	0.85	6.00E-25	1.07778	9.29E-09
rs2442732	rs115437294	6	31313042	HLA-B	intron	A/T	0.89	6.30E-14	1.09286	2.10E-10
rs709055	rs709055	6	31324151	HLA-B	exon	T/C	1.11	6.70E-11	0.93277	3.59E-08
rs12721827	rs12721827	6	31324210	HLA-B	exon	A/G	0.88	9.30E-13	1.07466	2.19E-07
rs1131163	rs1131163	6	31324888	HLA-B	exon	T/G	0.86	4.90E-26	1.07058	1.42E-08
rs2596544	rs139099016	6	31329291	HLA-B	flanking	A/T	1.2	2.60E-31	0.8996	8.22E-13
rs2428492	rs149497261	6	31329302	HLA-B	flanking	T/C	1.09	1.30E-10	0.93361	3.56E-09
rs9267068	rs9267068	6	31397521	HCP5	intron	A/G	0.73	1.30E-38	1.09286	3.85E-07
rs2855812	rs2855812	6	31472720	MICB	intron	T/G	1.17	3.00E-23	0.9352	1.22E-07
rs1265888	rs1265888	6	32066447	TNXB	intron	A/G	1.19	5.20E-22	0.90041	1.90E-11
rs915894	rs114346832	6	32190390	NOTCH4	exon	T/G	0.89	2.10E-17	1.07423	1.23E-08
rs2073045	rs115663894	6	32339548	C6orf10	exon	A/G	1.5	3.70E-151	0.92561	6.32E-08

### Expression of GAPPS

After identifying the 8 GAPPS as described above, we analyzed their expression patterns in different tissues by searching public databases. We studied their expression first in the brain regions under the assumption that it is likely the site for key SZ pathology and then, we also evaluated expression in the following immune-related cells: Epstein Barr virus (EBV)-transformed lymphocytes, spleen, and whole blood. The following brain regions were evaluated: amygdala, anterior cingulate cortex, caudate, cerebellar hemisphere, cerebellum, cortex, frontal cortex, hippocampus, hypothalamus, nucleus accumbens, putamen, spinal cord cervical c1, and substantia nigra. Of the 8 GAPPS, *HLA-C* and *HLA-B* were consistently expressed in all brain regions, while the expression of *TNXB, NOTCH4, MICB, PSORS1C1*, and *HCP5* was much lower. C6orf10 was not expressed in the brain. In EBV-transformed lymphocytes, spleen, and whole blood, the expression of *HLA-C*, *HLA-B*, and *HCP5* has been noted consistently, whereas *TNXB*, *NOTCH4*, and *MICB* are less consistently expressed in all three tissues. *PSORS1C1* has minimal expression in the lymphocytes and spleen and is not expressed in whole blood. C6orf10 is not expressed in any of the these tissues.^12^ Expression patterns were not used to further filter GAPPS.

### Interactome analyses

Analysis of the network of PPIs (or “interactome”) can reveal higher level functional relations among genes, which may not be apparent by analyzing the genes in isolation.^11^ For example, we recently studied the relation between SZ-risk genes that were identified through GWAS and those that were considered to be associated with SZ in pre-GWAS era, and showed that although they shared only one common gene, they had several common interactors, and that their interactomes shared common functional pathways.^11^ We constructed the interactomes of genes associated with RA as identified through GWAS (“RA genes”, “RA interactome”),^13^ and genes associated with SZ through GWAS (“SZ genes”, “SZ interactome”),^11, 14^ and analyzed the interconnections between them. We also constructed the interactome of GAPPS and analyzed how closely they are connected to the RA and SZ genes. To construct the interactomes, we included previously known PPIs and also novel PPIs that were discovered using our HiPPIP model.^11^ The novel PPIs were shown to be highly accurate based on computational and experimental evaluations as described in our prior work.^11^

**Fig. 1:**
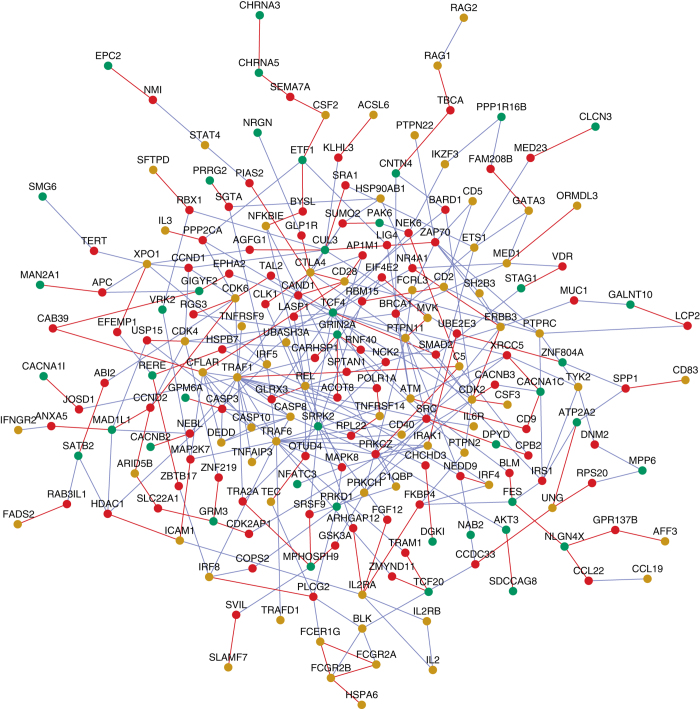
Novel interactors that connect RA and SZ genes: RA genes (*gold-colored nodes*) and SZ genes (*green nodes*) connect to each other either directly or through intermediate interactors (*red nodes*). Novel PPIs predicted with HiPPIP are shown as red lines (“edges”) and known PPIs as *blue edges*. Their inter-connections mediated by genes that have at least one novel interaction are shown here, whereas all PPIs including those mediated by known interactors are given in Supplementary File 2. Novel interactions that connect RA genes with each other and SZ genes with each other are also shown

The RA interactome consists of 98 RA genes and 1960 interactors, connected by 598 novel PPIs and 2232 known PPIs. Similarly, the SZ interactome consists of 77 SZ genes and 968 interactors, connected by 365 novel PPIs and 814 known PPIs. There are 316 genes in common between the RA and SZ interactomes, including direct PPIs between 7 RA genes and 6 SZ genes (Fig. 1 and Supplementary File 2), which is statistically highly significant (*p* < 10^−72^, hypergeometric distribution test). A subset of the interactome that highlights the inter-connections between RA and SZ genes mediated by novel PPIs is shown in Fig. 1. A complete list of PPIs that connect SZ and RA genes either directly or through a single intermediate interactor to each other are given in Supplementary File 2. This also includes a number of novel PPIs of RA genes, whereas novel PPIs of SZ genes were presented in our earlier work described above.^11^ The PPIs and the genes are clearly labeled as novel or known and also by their membership in the two interactomes.

**Fig. 2:**
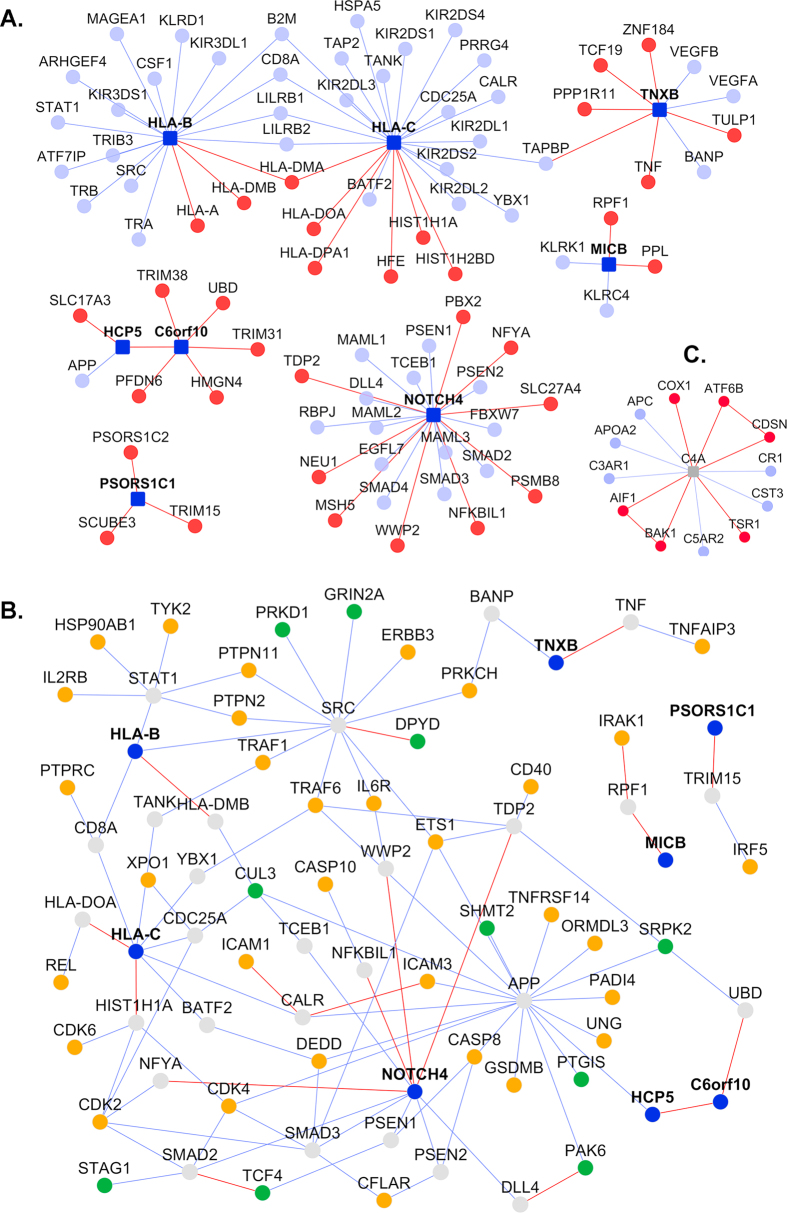
Interactome of genes associated with putative pleiotropic SNPs (GAPPS): **a** GAPPS interactomes: GAPPS (*dark blue square nodes*), novel interactors (*red* nodes) and known interactors (*blue nodes*), novel PPIs (*red lines* or “edges”) and known PPIs (*blue edges*). **b** The GAPPS interactome network is extended to show how its genes further interact with RA or SZ associated genes (*gold* and *green nodes*, respectively). GAPPS interactors that do not connect to RA or SZ genes are not shown here. **c** C4A interactomes, with legend same as in **a**

The interactome of the GAPPS (Fig. 2a) consisted of 8 GAPPS (*dark blue nodes*) connected to 33 novel interactors (*red nodes*) and 50 known interactors (*light blue nodes*) through 36 novel PPIs (*red lines* or “edges”) and 54 known PPIs (*blue edges*). One novel PPI shows that C6orf10 and HCP5 interact directly. Three of these genes had no known interactions except one PPI connecting HCP5 to APP, but we predicted 9 novel PPIs. Even for NOTCH4, HLA-B and HLA-C, which are well-studied genes with several known PPIs, we predicted additional novel PPIs for each. The GAPPS do not directly interact with any of the RA or SZ associated genes, but they connect to 32 RA genes (Fig. 2b: gold nodes) and 10 SZ genes (*green nodes*) through 27 common interactors (*grey nodes*).

**Fig. 3:**
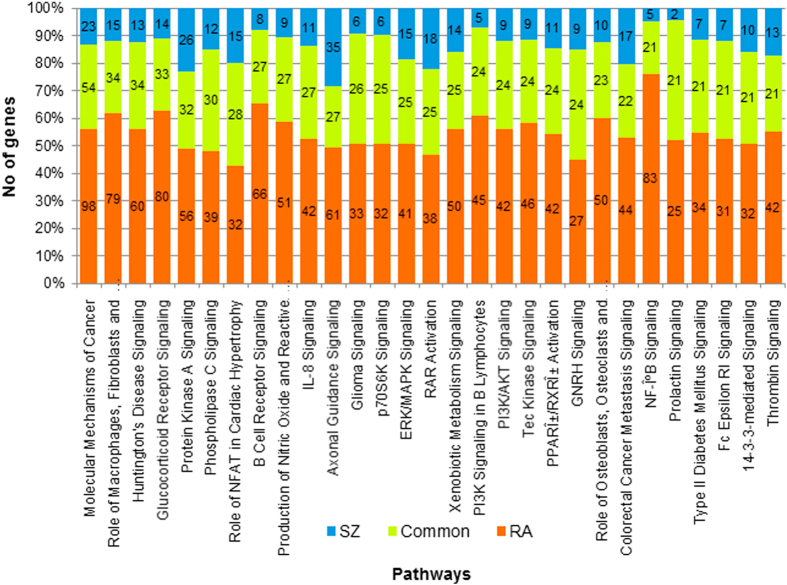
Common pathways associated with SZ and RA gene interactomes: Pathways associated with the interactome are computed with Ingenuity Pathway Analysis, which shows not only the significance of the association of the pathway but also the genes within the interactome that are associated with that pathway. Pathways are computed separately for SZ and RA gene interactomes. Shown here are the top 30 pathways in the SZ interactomes, along with number of genes associated exclusively with SZ interactome (*blue*), exclusively with RA interactome (*orange*) and common to both (*green*)

Next, we studied the pathways associated with the RA and SZ interactomes separately using Ingenuity Pathway Analysis® (IPA) suite (www.ingenuity.com). There are several pathways common to both the interactomes (Supplementary File 1). The commonality arises from not only the shared genes between the interactomes, but also from additional genes that are exclusive to either interactome (see selected pathways in Table 2). To highlight this aspect, we show in Fig. 3, the top 30 pathways associated with SZ interactome and the number of genes that are associated with each pathway that are exclusive and common to the two interactomes. For example, the *glucocorticoid receptor signaling* pathway is associated with 33 proteins from both interactomes, 80 proteins exclusive to RA interactome, and 14 proteins exclusive to SZ interactome.

**Table 2:** Selected pathways and the genes associated with them from the RA and SZ interactomes

Pathway	RA *p*-value	SZ *p*-va**lue**	Genes exclusive to RA Interactome	Genes common to RA & SZ Interactomes	Genes exclusive to SZ Interactome
Role of NFAT in Regulation of the Immune Response	6.3 E-28	3.0 E-07	NFATC2, NFKBIB, GNAO1, HRAS, ATM, NFKBIE, CD4, MAP2K1, CD247, IKBKAP, IKBKG, IKBKE, CD86, RAF1, CD79A, XPO1, FCER1G, FCER1A, PIK3CA, PIK3CD, PIK3CG, CSNK1D, MAPK1, PIK3R3, PIK3R2, NFKB2, CD80, MS4A2, CD28, PLCB4, FCGR2B, FCGR2A, GNG5, PRKCQ, GATA4, LYN, NFKBIA, IKBKB, ITK, SOS1, LCK, CSNK1E, GRB2, GNA12, HLA-DOA, CD79B, MEF2D, LAT, FCGR3A/FCGR3B, PPP3CB, CD3E, ATF2	GSK3A, NFATC3, MAPK3, SYK, JUN, FOS, FYN, CHUK, CALM1 (includes others), GNB2L1, PLCG2, PLCG1, AKT1, ZAP70, PIK3R1, LCP2, NFKB1, RELA, BTK	CSNK1A1, GNAS, GNB5, GNG10, GNB1, GSK3B, AKT3, HLA-DMB
IL-8 Signaling	3.98E-24	3.16E-13	BCL2L1, PTK2, MAP4K4, LIMK2, PIK3CD, HRAS, LIMK1, IKBKB, RHOC, MAP2K1, IKBKG, MAP2K4, FLT4, RAF1, EGF, FLT1, TEK, ATM, PIK3CA, RPS6KB1, PIK3CG, NFKBIB, MAPK1, PIK3R3, PIK3R2, IKBKE, IRAK2, IRAK3, IRAK1, KDR, ITGAM, PRKCI, ITGB2, ICAM1, CDH1, PRKCQ, RHOA, BRAF, CCND3, GNG5, GNA12, IRAK4	PRKCH, EGFR, AKT1, PTK2B, JUN, GNB2L1, MAPK3, PIK3R1, NFKB1, RELA, SRC, TRAF6, FOS, PRKCA, PRKCB, CHUK, PRKCG, PRKCD, PRKCZ, MTOR, LASP1, MAPK8, CCND1, CCND2, EIF4EBP1, PRKD1, BCL2	PTGS2, PRKCE, GNAS, GNB5, GNB1, PLD3, GNG10, RHOJ, AKT3, MAPK9, MYL9
CD28 Signaling in T Helper Cells	7.94E-27	2.04E-07	NFATC2, IKBKE, NFKBIB, ATM, NFKBIE, IKBKB, MAP2K1, CD247, IKBKG, MAP2K4, CD86, FCER1G, PIK3CA, PIK3CD, PIK3CG, GRAP2, PIK3R3, PIK3R2, PTPRC, NFKB2, MALT1, CTLA4, CD28, NFKBIA, CSK, PRKCQ, WAS, PTPN6, CD4, ITK, VAV1, PTPN11, CD3E, GRB2, IL2, HLA-DOA, CD80, LAT, PPP3CB, ACTR2, BCL10, LCK	NFATC3, FOS, SYK, CALM1 (includes others), FYN, CHUK, AKT1, PLCG1, JUN, ZAP70, PIK3R1, LCP2, NFKB1, RELA, MAPK8	ARPC3, ARPC1B, AKT3, PDPK1, MAPK9, HLA-DMB, CDC42
Natural Killer Cell Signaling	6.31E-25	5.50E-08	KLRC1, SH2D1B, HRAS, SH2D1A, MAP2K1, CD247, LAIR1, RAF1, FCER1G, INPPL1, PIK3CA, PIK3CD, PIK3CG, MAPK1, PIK3R3, PIK3R2, PRKCI, ATM, SHC1, NCR1, FCGR2A, PRKCQ, KIR2DL5A, PTPN6, INPP5D, NCK1, SOS1, VAV1, PTPN11, LCK, GRB2, KIR2DL4, LAT, FCGR3A/FCGR3B, SIGLEC7, VAV3	PRKCH, PRKCB, MAPK3, PRKCA, SYK, VAV2, FYN, PRKCD, PRKCG, PLCG2, PLCG1, PRKCZ, AKT1, ZAP70, PIK3R1, LCP2, PRKD1	PAK4, PAK6, AKT3, PRKCE, INPP5K
B Cell Receptor Signaling	6.31E-48	6.31E-12	BCL2L1, NFATC2, PTK2, CFL1, NFKBIB, PAG1, HRAS, NFKBIA, GAB2, NFKBIE, IKBKB, MAP2K1, RAP2A, IKBKG, IKBKE, PIK3CG, CD79B, ETS1, CD79A, MAP3K8, MAP2K4, INPPL1, ATF2, PIK3CA, PIK3CD, MAP3K4, MAP3K5, ELK1, PIK3R3, PIK3R2, RAF1, MAP3K14, LYN, MALT1, ATM, SHC1, CSK, RPS6KB1, CAMK2D, CD22, CAMK2G, FCGR2B, CREB1, FCGR2A, PAX5, MAP2K6, NFKB2, PRKCQ, GAB1, PTPN6, INPP5D, SOS1, VAV1, PTPN11, DAPP1, RASSF5, MAP3K11, GRB2, PTPRC, MAP3K3, MAPK1, PPP3CB, BCL6, VAV3, BCL10, CD19	NFATC3, CALM1 (includes others), MAPK14, PLCG2, PTK2B, MAP2K7, ABL1, EGR1, JUN, MAP3K7, MAPK3, PIK3R1, NFKB1, RELA, PRKCB, CAMK2A, CAMK2B, CHUK, MTOR, EP300, MAPK8, AKT1, SYK, VAV2, GSK3A, CREBBP, BTK	TCF3, INPP5K, GSK3B, PTEN, AKT3, PDPK1, MAPK9, CDC42

In another analysis using NextBio suite of tools,^15^ we analyzed gene expression patterns in schizophrenia and rheumatoid arthritis, and found a negative correlation between them: i.e., some genes that are over-expressed in one disorder are under-expressed in the other. For example, the gene expression data from neurons of SZ patients and synovial tissues of RA patients showed that there are 58 genes upregulated in SZ and downregulated in RA, and 48 genes are downregulated in SZ and upregulated in RA. We found 5 datasets that show a similar relationship between these diseases (Supplementary File 4). Overall, there were 369 such genes with opposite expression in the two diseases. Of these, 101 were found to among the SZ and RA interactomes, and pathway analysis showed that they are related to chemokine receptor signaling, signaling of IL-15, IL-12, IL-2, and IL-6 and Natural Killer Cell Signaling pathways, among others. Seven of them were found to be novel interactors of the SZ genes (ALDH6A1, FBLN1, MYL9, NKG7, RGS1, SETBP1, and SYNGR1), and twenty were novel interactors of RA genes (ARHGAP6, BMP4, CADM3, CYR61, DIO3, EFEMP1, GMPR, GNPDA1, IRF9, KCNMA1, MGST3, MX1, NET1, PEX6, RAB31, RAP2A, SGCE, SPP1, UAP1, and ZFP36L1); of these, EFEMP1 and SPP1 are also known interactors of SZ. Some studies identified SPP1 as an RA-susceptibility gene and hence this novel interactor may have a role in RA pathogenesis. These interactome analyses further strengthen the link between rheumatoid arthritis and schizophrenia, and also highlight the functional significance of novel predicted interactors in the disease processes.

We used this 369 gene signature to query large scale perturbagen signatures (L1000 profiles) from the NIH’s Library of Integrated Network-based Cellular Signatures (LINCS—www.lincscloud.org) to identify small molecules that could be potentially therapeutic for SZ or RA. Among the top compounds were both known and investigational compounds used for RA (e.g. bortezomib) or SZ (e.g., trifluoperazine, ropinirole) (Supplementary File 5). By studying the expression signatures associated with gene knockdown (i.e., gene perturbagens) in LINCS database, we found that there are 150 genes whose knockdown signature correlates with the observed signature of differential expression. Of these 150, about one-third were included in the interactomes.

## Discussion

Mounting data supports the pathophysiological importance of neuroinflammation in SZ.^16–25^ SZ postmortem studies note activated microglia/macrophages,^16, 20, 26^ elevated expression of inflammatory markers in the prefrontal cortex neurons^27^ and vasculature,^28^ and autoantibodies against frontal,^29^ cingulate,^29–31^ hippocampal cortices,^30^ and glutamate receptors.^32^ Autoimmune disorders may elevate the risk for SZ both independently^33, 34^ and in combination with infections.^33^ A meta-analysis noted elevated peripheral blood inflammatory cytokines in SZ compared to healthy controls.^35^ Genome-wide association studies replicate the association of variants in the major histocompatibility complex region (where a large number of immune genes are located) with SZ.^36–39^ Non-steroidal anti-inflammatory drugs may reduce psychotic symptom severity.^40–42^ We noted anatomical dysconnections^43^ and increased neuropil pruning^44^ associated with peripheral inflammatory markers suggesting possible mechanisms through which inflammation may underlie schizophrenia pathogenesis. While there is ample evidence for such correlations, to our knowledge, there are no biological data that directly support the relation between SZ and RA. We have utilized an in silico cross-disciplinary approach to determine the possible focal points of such relation.

We initially identified GWAS SNPs to find those that show apparent pleiotropic effects on risk for SZ and RA based on the inverse epidemiological relationship between the two diseases and prior indicated negative correlations for genetic risk.^8, 10^ The SNP-based analyses identified 29 SNPs, of which 11 were localized to intergenic regions and 18 were localized to genes including their flanking regions. As one of the goals of our study was to identify genes with high probability of involvement in SZ pathology that could be investigated further in subsequent studies, we conducted additional in silico analyses of 18 SNPs within genes or their flanking regions (Table 1), recognizing that the SNPs in intergenic regions continue to be of interest and deserve functional analyses. Through the location of the 18 putative pleiotropic SNPs, we identified GAPPS and constructed their interactomes. In parallel, and independent of the hypotheses based on pleiotropic effects, we carried out interactome analyses of RA and SZ genes to find network and pathway relations between the two diseases. To our knowledge, such analyses relating SZ and RA have not been conducted before.

All the SNPs with putative pleiotropic effects were localized to the extended HLA region. Associations of SZ and RA in this region have been known for over four decades and have also been confirmed through GWAS.^45, 46^ As there is extensive LD in this gene-rich region and the risk attributable to individual variants is relatively small, it has been difficult to identify primary risk variants. Recently, a portion of the risk in the HLA region has been attributed to relatively frequent copy-number variation (CNV) spanning the C4A-C4B complement genes, and the gene expression analysis of post-mortem tissues from several brain regions indicated that the C4A gene is over expressed among patients with schizophrenia.^47^ These analyses also indicate additional, independently acting risk variants in the HLA region.^47^ Similarly, our analyses pointed to additional SNPs in HLA that should be explored further. To enable functional studies of C4A gene, we computed the interactome of C4A, and found that it has 6 novel interactors (Fig. 2c). C4A shares two common interactors (APC and ATF6B) with RA genes and one common interactor (APC) with SZ genes.

As the precise functional effects of the putative pleiotropic SNPs is unknown, we sought genes that might be impacted by such variation. Our analyses identified eight GAPP genes, of which HLA-B and HLA-C appear to have the greatest amount of published data relating to SZ or RA pathogenesis. Previous studies have shown genetic associations of HLA-B in both rheumatoid arthritis and schizophrenia. Allele HLA B27 has been associated with several types of arthritis disorders such as ankylosing spondylitis, reactive arthritis and psoriatic arthritis.^48^ Other studies have shown that similarity in certain regions of the HLA-B gene between the mother and the daughter give an increased risk of schizophrenia to the daughter.^49^ Aside from genetic associations, HLA-B may play a role in the two diseases by its involvement in the natural killer cell pathway. Natural killer cells (NKCs) are found to have low activity per cell in rheumatoid arthritis, as opposed to a high activity per cell found in schizophrenic patients.^50, 51^ However, other studies indicate that the lower activity is due to a lower number of circulating NKCs. NKC activity in schizophrenia patients, on the other hand, is elevated. Homozygosity in multiple alleles in the HLA-B gene is thought to lower NKC activity that has been associated with rheumatoid arthritis.^52–54^ The HLA-C protein is thought to act as a ligand for the killer immunoglobulin receptors found on NKC, thus acting as an NKC inhibitor and regulating their activity.^55^ Some polymorphisms in HLA-C are thought to cause a decreased risk of rheumatoid arthritis, while other studies indicate that polymorphisms in this gene cause an increased risk of schizophrenia.^56, 57^ The pleiotropic associations with HLA-C polymorphisms, the opposite activity levels of NKCs in the two diseases and their association with HLA-B, makes these genes prime candidates for further research. Among the remaining genes, *MICB* encodes for a stress-induced protein that is a ligand for the NKG2D Type 2 receptor, which is located on NKCs, CD8 alpha/beta T cells, and gamma/delta T cells. *HCP5* denotes the HLA complex protein P5, and while located in the HLA region, it is not similar in sequence to the other HLA genes. It is more similar to the human endogenous retroviruses HERV-L and HERV-16. Tenascin XB (TNXB) is a member of the extracellular matrix glycoproteins and several genetic association studies have indicated TNXB SNPs as risk factors for SZ, but similar associations with RA have not been reported, to our knowledge. NOTCH4, a transmembrane protein mediating the Notch signaling pathway, regulates interactions between adjacent neurons. Multiple genetic association studies have associated NOTCH4 with schizophrenia and rheumatoid arthritis, but their functional implications are uncertain.^58, 36^

Using interactome analysis, we found that the eight GAPPS share common interacting partners with RA and SZ genes (Fig. 2b). It also showed that even though RA and SZ do not share common risk genes beyond the HLA region, they have 316 common interacting partners through PPIs (Fig. 1 and Supplementary File 2). There are even direct PPIs between RA and SZ genes: 7 RA genes and 6 SZ genes interact with each other. Two of the RA genes, namely CSF2 and UNG, are novel interactors of SZ genes. One of the subunits of CSF2 (CSF2RB) is essential for IL3 signaling which is involved in schizophrenia pathology.^59^ Similarly, the SZ genes ATP2A2 and ETF1 are novel interactors of RA genes.

The parallel analyses of the SZ and the RA interactomes indicated that there are several pathways that are common to, and significantly associated with both the disorders, though it is likely that some of these pathways are disrupted in other disorders (Fig. 3 and Supplementary File 2). We found several pathways that are associated with immune function and inflammation. For example, pathways such as *role of NFAT in regulation of immune response* has 43 common genes, *IL-8 signaling* has 27 genes, *CD28 signaling in T-Helper cells* has 15 genes, *natural killer cell signaling* has 17 genes, *crosstalk between dendritic cells and natural killer cells* has 6 genes, *NF-kB signaling* has 21 genes and *B cell receptor signaling* has 27 common genes. Prior studies suggested that interleukins may be associated with schizophrenia pathology.^60^ The *NF-kB signaling* pathway may also have an important role because NF-kB molecule has key role in immune response regulation and is associated with RA pathology; it has also been implicated in synaptic plasticity and memory, which are commonly altered in schizophrenia patients.^61, 62^ The PPIs, including novel PPIs that we predicted, highlight how the RA and SZ genes connect to pathways that are of interest in the biology of both the diseases (examples shown in Table 2). All these pathways have highly significant associations with both interactomes, though it is recognized that some of these pathways are likely involved in other disorders (Supplementary File 2).

Some limitations of the present work should be noted. In addition to pleiotropy, there are undoubtedly other mechanisms for the inverse prevalence of SZ and RA that need to be explored. Our studies were solely in silico, and need to be verified through experiments. Genetic risk variants with pleiotropic effects, which were not within genes or their flanking regions, were not explored further (Supplementary File 1). Other variants such as a CNV on C4A could not be analyzed in detail.^47^ The functional implications of the novel PPIs predicted for C4A (Fig. 2c), especially those that have links to both RA and SZ genes, should be studied further.

It could be argued that instead of evaluating effects of risk SNPs in function of their respective genes, one ought to evaluate their effects on the overall “output” of the respective pathways. Our studies motivate these and similar studies. A related question that would need to be addressed is the impact of such changes on the “end organs” for SZ and RA, i.e., the brain and the joints, respectively.

In conclusion, we integrated epidemiological, GWAS, gene expression and proteomic data to identify genes and pathways with potential pathogenic relevance for both SZ and RA. We recommend avenues for further functional analyses based on these hypotheses.

## Methods

### Identifying SNPs with putative pleiotropic effects

**Fig. 4:**
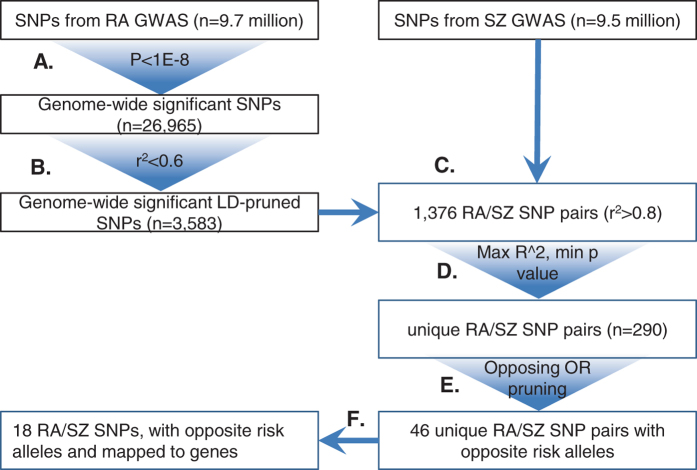
Overview of methods to identify SNP pairs with pleiotropic effects: Detailed flowchart representing all of the steps in the thresholding analysis starting with the genome-wide SNPs tested for RA and SZ associations

The overview of the procedure for identifying SNPs with putative pleiotropic effects is shown in Fig. 4. We used publicly available SNP level summary statistics from large scale GWAS of schizophrenia and rheumatoid arthritis. The SZ dataset included results from 36,989 cases and 113,075 controls for approximately 9.5 million SNPs.^14^ The RA dataset included results from 29,880 cases and 73,758 controls for approximately 9.7 million SNPs.^13^

The analysis started with the selection of candidate RA SNPs, where our goal was to prepare a list of SNPs that are not highly correlated with each other and would individually confer statistically significant risk at genome-wide levels. We combined linkage disequilibrium (LD) pruning with p-value thresholding (*p* < 10^−8^) on the RA dataset using ‘Swiss’ software (www.github.com/welchr/Swiss). LD between pairs of SNPs was calculated based on 1000 Genomes data (phase II, Caucasian ancestry sample),^63^ with a threshold of *r* ^2^ ≤ 0.6 to identify a relatively independent set of RA SNPs. Each of these highly significant and low-LD RA SNPs was paired with each of the SZ SNPs in the initial GWAS list. Out of these, we selected the pairs that had an LD value of r^2^ ≥ 0.8 between them, resulting in 1376 SNP pairs. When multiple SZ SNPs matched the same RA SNP, the pair with the highest LD between them and the lowest *p*-value for the SZ SNP was chosen; this resulted in 290 pairs of SNPs.

The odds ratios (OR) and reference alleles of the resulting pairs were then examined as given in the original summary statistics, to identify those which had opposite odds ratios (i.e., where SNP of one disorder had OR < 1 and the SNP of the other disorder had OR > 1).^64^ This resulted in 46 (*r*, *z*) SNP pairs.

In the analysis thus far, for the SNP pair (*r*, *z*), SNP *r* was picked for its effect on RA and z for its effect on SZ; but we also verified that the odds ratio of *r* for RA and *r* for SZ are in opposite directions, and vice versa for *z*. This was found to be applicable only where *r* and *z* referred to the same SNP but not otherwise. Thus, at the end of the analysis, there were 29 individual SNPs with opposing effects on the two diseases.

These SNPs were mapped to gene boundaries including 5000 base upstream and downstream flanking regions, as given in the Known Canonical Genes track of UCSC Genome Browser for the human genome build hg19 (GRCh37).^65^ This resulted in 18 SNPs. Only these 18 SNPs that were localized within gene boundaries were considered for further analysis. The genes within which these 18 SNPs were located are referred to as GAPPS.

### Expression analysis of GAPPS

The GTEx browser was used to analyze tissue expression for the 8 GAPPS.^12^

### Interactome construction and analysis

The interactomes were assembled by collecting known PPIs from the Human Protein Reference Database^66^ and Biological General Repository for Interaction Datasets,^67^ and by computing novel PPIs using the HiPPIP model that we developed.^11^ The predicted PPIs have been shown to be highly accurate by computational evaluations and experimental validations of a few PPIs in our earlier work.^11^ Interactome figures were created using Cytoscape.^68^ Pathways associated with proteins in RA interactome and SZ interactome were collected separately using Ingenuity Pathway Analysis® suite (www.ingenuity.com). All the proteins including candidates, known and novel interactors that are present in each interactome were loaded into IPA suite, which returns all the pathways associated with any of the genes and the list of those genes, and the statistical significance of association computed with Benjamini-Hochberg correction for multiple testing, a widely used method to control the rate of false discoveries in statistical hypothesis testing. A corrected *p*-value *P* can be interpreted as an upper bound for the expected fraction of falsely rejected null hypotheses among all functions with *p*-values smaller than *P*.^69^ Supplementary File 3 shows all pathways associated with any gene(s) from the interactomes irrespective of the statistical significance of association so that readers can use the supplementary data to not only identify significantly associated pathways (by choosing a stringent *p*-value threshold) and also to query individual genes for their functional/pathway associations irrespective of that pathway being significant in the interactome; for example, novel interactors of SZ genes PRKAG1, PRKAR1B and two other known interactors are found to be involved in *sonic hedgehog signaling* pathway, which may be useful to know although that pathway is not statistically significant in the overall interactome.

The list of pathways associated with each of the two interactomes were merged using a computer program to present clearly which of the two interactomes the genes of associated with the pathway belonged to, how many genes from each interactome were associated with that pathway, and what the B-H corrected *P*-value of significance was for that pathway with each interactome. While a selected few pathways are shown in Table 2, a full list is given in Supplementary File 3. The pathways shown in Table 2 and Fig. 3 are statistically significant (*p*-value < 10–6), whereas Supplementary File 3 shows all pathways associated with any gene(s) from the interactomes irrespective of the statistical significance of association.

NextBio is a suite of tools that enables the study of correlated effect of diseases and/or drugs on gene expression using publicly available gene expression data.^15^ We used this in an independent analysis to identify genes that were overexpressed in one disease (either RA or SZ) and under-expressed in the other. We queried the genes in this RA–SZ reciprocal expression against the LINCS database (http://www.lincscloud.org/), a massive catalog of differential gene-expression profiles of human cells resulting from treatment with chemical and genetic perturbagens. With this, we identified gene knockouts and chemical compounds which result in differential gene expression pattern that correlates with RA (anti-correlation with SZ) or correlates with SZ (anti-correlation with RA).

## Electronic supplementary material

Supplementary File 1

Supplementary File 2

Supplementary File 3

Supplementary File 4

Supplementary File 5
